# Adoptive cellular therapies: the current landscape

**DOI:** 10.1007/s00428-018-2484-0

**Published:** 2018-11-23

**Authors:** Maartje W. Rohaan, Sofie Wilgenhof, John B. A. G. Haanen

**Affiliations:** Department of Medical Oncology, The Netherlands Cancer Institute, Plesmanlaan 121, 1066 CX Amsterdam, The Netherlands

**Keywords:** Adoptive cell therapy, Immunotherapy, Tumor-infiltrating lymphocytes, T cell receptor, Chimeric antigen receptor

## Abstract

For many cancer types, the immune system plays an essential role in their development and growth. Based on these rather novel insights, immunotherapeutic strategies have been developed. In the past decade, immune checkpoint blockade has demonstrated a major breakthrough in cancer treatment and has currently been approved for the treatment of multiple tumor types. Adoptive cell therapy (ACT) with tumor-infiltrating lymphocytes (TIL) or gene-modified T cells expressing novel T cell receptors (TCR) or chimeric antigen receptors (CAR) is another strategy to modify the immune system to recognize tumor cells and thus carry out an anti-tumor effector function. These treatments have shown promising results in various tumor types, and multiple clinical trials are being conducted worldwide to further optimize this treatment modality. Most successful results were obtained in hematological malignancies with the use of CD19-directed CAR T cell therapy and already led to the commercial approval by the FDA. This review provides an overview of the developments in ACT, the associated toxicity, and the future potential of ACT in cancer treatment.

## Introduction

In the past few decades, the potency of the immune system in the development and treatment of cancer has been a major focal point of research [1]. Although targeted therapy and immunotherapy with immune checkpoint blockade have greatly improved the survival of, amongst others, melanoma and non-small cell lung cancer patients, a large proportion of patients still develop disease progression upon these therapies [2, 3]. Adoptive cell therapy (ACT) may provide an additional treatment option for these patients and comprises the intravenous transfer of either tumor-resident or peripheral blood modified immune cells into cancer patients to mediate an anti-tumor function. Currently, ACT can be classified into three different types with each their own mechanism of action, namely ACT with tumor-infiltrating lymphocytes (TIL), ACT using T cell receptor (TCR) gene therapy, and ACT with chimeric antigen receptor (CAR) modified T cells [4]. The use of other immune cell types such as natural killer cells as a basis for cell therapy is also an area of current research. However, many hurdles have to be overcome in order for this to be an effective anti-cancer treatment [5] and lie beyond the scope of this review.

The first studies with TIL were performed by Rosenberg and coworkers at the Surgery Branch in the National Institutes of Health (SB, NIH, Bethesda, Maryland, US), where TIL were grown from different murine tumors and showed anti-tumor activity in vivo [6]. The current TIL therapy consists of ex vivo expansion of TIL from resected tumor material and adoptive transfer into the patient following a lymphodepleting preparative regimen and subsequent support of interleukin-2 (IL-2). With this regimen, remarkable objective tumor responses of around 50% have been achieved in patients with metastatic melanoma in several phase I/II clinical trials [7–9]. After the successes seen with TIL in melanoma patients, the production of TIL from other solid tumor types has also been studied. Up until now, it has been possible to grow out TILs from non-melanoma tumor types such cervical cancer [10], renal cell cancer [11], breast cancer [12], and non-small cell lung cancer [13] with varying rates of tumor reactivity.

**Fig. 1:**
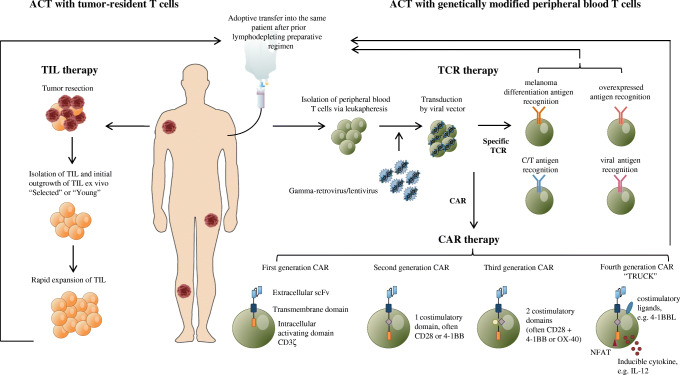
Schematic overview of the processes for adoptive cell therapy (ACT) of tumor-infiltrating lymphocytes (TIL), ACT with T cell receptor (TCR) gene therapy and ACT with chimeric antigen receptor (CAR)-modified T cells. In ACT with TIL, tumor-resident T cells are isolated and expanded ex vivo after surgical resection of the tumor. Thereafter, the TILs are further expanded in a rapid expansion protocol (REP). Before intravenous adoptive transfer into the patient, the patient is treated with a lymphodepleting conditioning regimen. In ACT with genetically modified peripheral blood T cells, TCR gene therapy and CAR gene therapy can be distinguished. For both treatment modalities, peripheral blood T cells are isolated via leukapheresis. These T cells are then transduced by viral vectors to either express a specific TCR or CAR, respectively

Next to the naturally occurring TILs in tumors and the thereupon-based treatment options, peripheral blood T cells can be isolated and genetically modified in vitro to express TCRs that target specific tumor antigens for the use of ACT. With the use of this method, large pools of tumor specific T cells can be generated [14, 15], with potent anti-tumor activity and objective clinical responses observed in up to 30% of treated patients [16]. For the recognition by the modified TCR, antigen presentation via the major histocompatibility complex (MHC) is required. However, it is well known that many cancer types can escape T cell-mediated immune responses by downregulation or loss of their MHC expression [17]. To circumvent the need for the presence of MHC on tumor cells for the recognition by tumor-specific T cells, artificial receptors such as CAR molecules have been developed. ACT with CAR-modified T cells holds the capacity of the same effector function as TCR-modified T cells, but independently of MHC expression [18]. Besides the use of protein antigens, other antigens such as carbohydrates [19] or glycolipid antigens [20, 21] have also been explored. Impressive clinical responses have already been seen in hematological malignancies with CD19-specific CAR T-cells [22], which led to the exploration of using CAR therapy in solid tumors as well [23]. Table 1 summarizes these three different treatment modalities of ACT and Fig. 1 shows an overview of the process for adoptive cell therapy with TIL, TCR gene therapy and CAR-modified T cells.

**Table 1:** Overview of treatment modalities of adoptive cell therapy

	TIL	TCR	CAR
First evidence of clinical benefit	1994 [24]	2006 [25]	2013 [26]
Production method	Isolation of T cells from tumors and expansion ex vivo	Isolation of peripheral T cells via apheresis and ex vivo transduction with a TCR against tumor antigen	Isolation of peripheral T cells via apheresis and ex vivo transduction with a CAR against tumor antigen
Target	MHC-peptide complex	MHC-peptide complex	Non-MHC cell surface proteins
Lymphodepleting preparative regimen	Yes	Yes	Yes
Supportive IL-2	Yes	Varying	No
Specificity	Polyclonal	Monoclonal	Monoclonal
Main toxicity	Lymphodepleting regimen IL-2 mediated (chills, fever, edema) Seldom autoimmune	Lymphodepleting regimen “On-target, off-tumor” CRS	Lymphodepleting regimen “On-target, off-tumor” CRS Neurological
Restrictions	Complex Heterogeneous infusion product	MHC-restricted Currently not yet tumor-specific Toxicity	Currently only effective for treatment of hematological malignancies Toxicity

*CAR* chimeric antigen receptor, *CRS* cytokine release syndrome, *IL-2* interleukin-2, *MHC* major histocompatibility complex, *TCR* T cell receptor, *TIL* tumor-infiltrating lymphocytes

Although most studies with ACT in solid tumors have been performed in melanoma, the role of ACT in the treatment of other tumor types is growing. Recently, an overview of initiated trials conducted with ACT since May 2015 was published by Fournier et al. [27], where an impressive 121 new clinical trials were described (including ACT in non-solid tumors). This illustrates the need for up-to-date knowledge on ACT in this quickly developing field. The aim of this review is to give a comprehensive overview of the previous developments and the current status of ACT, as the potential of ACT as treatment modality in cancer continues to rise.

## Adoptive cell therapy with tumor-resident T cells

The presence of TIL in neoplastic tissue is thought to indicate an anti-tumor immune response by the host and correlates with clinical outcome in several tumor types, especially in melanoma [28, 29]. Dr. S. Rosenberg (SB, NIH, Bethesda, Maryland, US) was the first to demonstrate the anti-tumor activity of TIL in vivo in murine models in the 1980s of the past century [6]. Combining T cell growth factor IL-2 with the TIL infusion product resulted in a greater therapeutic potency of TIL compared to lymphokine-activated killer (LAK) cells produced from peripheral blood lymphocytes in the presence of IL-2 in mice with metastases from various tumor types. Addition of cyclophosphamide to TIL and IL-2 further potentiated the anti-tumor effect of TIL [30]. These early murine studies formed the basis for the original and still most commonly used TIL treatment protocol.

In the original treatment protocol of TIL in metastatic melanoma, patients underwent resection of one or more metastases with a total diameter of at least 2–3 cm. The resected tumor was fragmented or enzymatically digested and subsequently cultured in the presence of IL-2, which resulted in proliferation of TIL. This initial outgrowth phase took approximately 14 days. Once culture consisted mostly of CD3^+^ T cells, their specificity was tested during a short culture in the presence of an autologous or HLA-matched tumor cell line by quantification of interferon-γ (IFN-γ) [7]. This selection step, however, was time-consuming and complex. Follow-up studies showed that TIL production without this pre-selection for tumor reactivity, so-called “young TIL,” resulted in comparable clinical responses [31, 32] and became the current standard treatment protocol. At least 50 × 10^6^ TILs from this initial outgrowth phase are required to be further expanded in a rapid expansion protocol (REP) in the presence of a soluble anti-CD3 antibody, IL-2 and irradiated allogeneic or autologous feeder cells. During this 14 days lasting expansion phase, up to approximately 1 × 10^11^ cells are obtained. These TILs are harvested and prepared for infusion into the patient [33]. Prior to infusion, patients will undergo lymphodepleting, but non-myeloablative (NMA) chemotherapy consisting of 2 days intravenous (i.v.) cyclophosphamide (60 mg/kg) followed by 5 days fludarabine (25 mg/m^2^). Shortly after infusion of TIL, patients receive i.v. high-dose (HD) bolus IL-2 (720,000 IU/kg every 8 h until maximal tolerance) [7, 24, 34]. Subsequent support with IL-2 is thought to further enhance the survival and clinical efficacy of TIL [35].

Multiple studies have been performed evaluating the role of preconditioning lymphodepleting regimens. Lymphodepleting regimens cause a short, but deep lymphopenia and neutropenia, with full bone marrow recovery within 7–10 days, not requiring hematopoietic stem cell support [36]. Murine models had shown that response rates upon TIL improved after prior lymphodepletion by total body irradiation (TBI) or chemotherapy [30, 37]. These models showed that depletion of endogenous lymphocytes created “physical space,” resulted in less competition for homeostatic cytokines IL-7 and IL-15 [38] and removed immunosuppressive lymphoid and myeloid populations [39]. Incorporating an intensified lymphodepleting regimen by combining cyclophosphamide/fludarabine with either 2 Gy or 12 Gy TBI, improved the overall response rate from 49% to respectively 52% and 72% in small cohorts of 25 metastatic melanoma patients. Significantly higher levels of IL-7 and IL-15 were measured in patients treated with the intensified lymphodepleting regimen (*p* = 0.02 and *p* = 0.005 respectively). However, this intensified regimen did result in more acute toxicity and prolonged organ dysfunction and required hematopoietic stem cell support for bone marrow recovery [34]. In a randomized controlled phase II trial by the NIH in 2016, the clinical benefit of addition of 12 Gy TBI to NMA chemotherapy could not be reproduced [40]. A currently recruiting phase II trial conducted by the Sheba Medical Center, Israel, evaluating the effect of a reduced intensity lymphodepleting regimen prior to TIL is expected to give further insight into the optimal preparative regimen (NCT03166397).

Most studies conducted with TIL have been performed in patients with advanced (cutaneous) melanoma, but the place of TIL in the current standard treatment protocol is still being investigated. Promising clinical responses have been observed in patients with metastatic uveal melanoma [41] and may provide a novel treatment modality for this group of patients, as no standard treatment is available yet. The currently recruiting phase III randomized clinical trial comparing ipilimumab to TIL treatment (NCT02278887) should provide more evidence for the role of TIL as anti-tumor treatment in metastatic melanoma. Successful production of TIL was also achieved in renal cell carcinoma [11], breast cancer [12], cervical cancer [10], gastrointestinal cancers [42, 43], cholangiocarcinoma [44], pancreatic cancer [45], head and neck cancer [46], ovarian cancer [47], and non-small cell lung cancer [13]. However, the antitumor reactivity of TIL obtained from these other solid tumors still remains a challenge. It has been demonstrated that TILs isolated from melanoma show consistent antitumor reactivity [48] and that high mutational load and high neo-antigen rates are significantly associated with clinical benefit upon TIL therapy in patients with metastatic melanoma [49]. The production and reactivity of TIL products from other solid tumor types is highly variable, most likely as a result of heterogeneity in mutational and neo-antigen burden, in lymphocytic infiltration with variations of CD4^+^ and CD8^+^ T cells, in myeloid infiltrate composition and other as yet unknown factors [50]. Further research is needed in this field in order to potentiate TIL treatment for other solid tumors.

## Adoptive cell therapy with genetically modified peripheral blood T cells

Antigen receptor gene engineering with TCRs or CARs is a developing and promising new anti-tumor modality [15]. In contrast to TIL, peripheral blood lymphocytes are enhanced to create anti-tumor reactivity by transduction with tumor-specific receptors. This modification can be performed with relevant tumor-reactive TCR for TCR gene therapy or synthetic antibody-based receptors for CAR T cell therapy [36].

### TCR gene therapy

In TCR gene therapy, tumor antigen recognition is achieved by the introduction of a novel T cell receptor into T cells. Autologous T cells are redirected to recognize tumor antigens by engraftment of genes encoding TCR-α and β chains. TCR-modified T cells exert antigen recognition in an MHC-dependent manner [23, 51]. Examples of targetable antigens are tissue-specific antigens like melanoma differentiation antigens, cancer/testis (C/T) antigens, overexpressed antigens, and viral antigens. In most clinical trials, peripheral blood T cells for genetic modification are obtained via leukapheresis and are transduced by gamma-retroviral or lentiviral vectors that incorporate the TCR genes into the host genome, which results in high-level expression of the introduced TCR [36, 52, 53]. Other means of genetic engineering that are currently in development include the transposon/transposase system, such as Sleeping Beauty [54], or Crispr/Cas9 based technology [55]. These technologies do not require the production of lenti- or gamma-retroviral vectors, and may therefore provide a more flexible and cheap platform. As in ACT with TIL, most clinical protocols with TCR gene therapy have incorporated preconditioning of the patient with a lymphodepleting regimen prior to T cell infusion, to facilitate engraftment and expand the lifespan of the modified T cells. In addition, IL-2 administration following T cell infusion has been used [56].

The first evidence of the feasibility and clinical potency of TCR gene therapy targeting the melanoma differentiation antigen MART-1, present in approximately 80–95% of melanomas [57, 58], was demonstrated in 17 patients with progressive metastatic melanoma. Gene transfer efficiencies of 17–62% were achieved and an objective partial tumor response was seen in two (13%) of the treated patients [25]. In a subsequent clinical trial, 36 patients with metastatic melanoma were treated with high-avidity TCRs targeting the melanoma differentiation antigens gp100 or MART-1 and objective tumor responses were observed in 19% and 30% respectively [16].

Next to melanoma differentiation antigens as a target for TCR gene therapy, notable clinical responses have also been achieved when targeting C/T antigens. The genes encoding these antigens are normally expressed during embryogenesis, but are epigenetically silenced later in life, except in spermatocytes. Cancers oftentimes aberrantly re-express these genes, hence the name C/T antigens. These antigens include NY-ESO-1, MAGE-family, and SSX [59]. NY-ESO-1 is expressed in up to 52% of melanomas [60], 82% of neuroblastomas [61], 80–100% of synovial sarcomas and mixoid and round cell liposarcomas [62], 43% of ovarian cancer [63] and to a lesser extent in multiple other tumor types. Because of its restricted expression in normal tissues in combination with a widespread expression in cancer, NY-ESO-1 has been frequently used as a target in TCR gene therapy. In a phase II clinical trial, 5/11 (45%) patients with progressive advanced melanoma and 4/6 (67%) patients with synovial sarcoma had an objective tumor response after infusion of 1.6–130 × 10^9^ retrovirally transduced autologous T cells with a NY-ESO-1 targeting high affinity TCR [64]. Similar response rates have been observed when targeting MAGE-A3, which is expressed in 62% of melanomas [65]. In a clinical phase I/II trial, nine pretreated patients with either advanced melanoma (*n* = 7), synovial sarcoma (*n* = 1), or esophageal cancer (*n* = 1) were treated with anti-MAGE-A3 TCR gene-modified T cells in a dose-escalating manner. The first three patients were treated with 3 × 10^10^ transduced cells and the remaining patients were treated with 1 × 10^11^ transduced cells, with a transduction efficiency of 70% (CD8^+^ T cells). Of these patients, five (56%), including four melanoma patients and the single synovial sarcoma patient, had an objective tumor response [66].

Tumor regression has also been seen in patients with T cells targeting carcinoembryonic antigen (CEA), which is overexpressed in colorectal adenocarcinoma but is also present in normal epithelial cells [67]. Three patients were treated with 2 × 10^8^–4 × 10^8^ CEA reactive-TCR transduced T cells, and one of these patients achieved a partial response. However, all 3 patients developed a severe transient colitis [68].

Viral antigens such as human papillomavirus (HPV) as possible targets for TCR therapy have also been explored for HPV-associated epithelial cancers including cervical, oropharyngeal, vulvar, vaginal, anal, and penile cancer. T cells engineered with the TCR recognizing the HPV-16 E6 epitope from metastatic anal cancer also showed recognition of HPV-16 positive cervical and head and neck cancer cell lines and this epitope may thus be a potential target for TCR therapy [69].

Transduction of T cells in an early differentiation stage (central memory CD8^+^ T cells) seems to result in greater anti-tumor responses when combined with tumor-antigen vaccination and exogenous IL-2 in preclinical murine models [70]. It has also been shown that the cytokines IL-7 and IL-15, which play a role in the development of central memory T cells in vivo, favor the generation of this T cell subset in culture systems [70, 71]. In most cancer immunotherapy studies, CD8^+^ T cells have been the main focus because of their known strong cytolytic capacity [72]. However, recent evidence shows that CD4^+^ T cells also exert anti-tumor efficacy [44] and CD4^+^ T cells have already been part of the TCR infusion product used in early clinical trials [16, 64, 66]. In a validated good manufacturing practice (GMP) production process, CD4^+^ as well as CD8^+^ T cells are retrovirally transduced and expanded in the presence of IL-7 and IL-15 in combination with anti-CD3/CD28 bead selection and activation [73]. This protocol is being evaluated in a phase I/IIa trial in HLA-A:*02-01 MART-1 positive patients with advanced melanoma, including patients with uveal melanoma (NCT02654821), and should provide more insight into the feasibility and safety of TCR therapy in melanoma.

In summary, these results demonstrate that TCR therapy can be a potent anti-tumor treatment in various cancer types. However, as the antigens explored up until now are not solely expressed by the tumor, the identification of antigens restricted to tumors is essential to further increase the efficacy and safety of TCR therapy.

### CAR T cell therapy

CARs are hybrid receptors and are currently genetically constructed to contain a scFv of a monoclonal antibody as the antigen-binding extracellular domain, an intracellular CD3ζ chain as the TCR signaling domain and an additional co-signaling domain, mainly CD28 and 4-1BB (CD137) or others, to deliver co-stimulation [23, 74]. Multiple methods to transfer CARs to T cells have been developed, but most commonly used is transfer by retroviral infection, which has proven to be efficacious and safe [75]. Induction of cytotoxic activity of the manufactured T cell is a result of antigen-binding to the scFv, leading to downstream signaling through phosphorylation of CD3ζ and additional signaling cascades via the co-stimulating domains [76], similar to signaling following T cell activation through the TCR complex. Unlike TCR gene therapy, CAR T cells show target recognition in an MHC-independent manner, as was first demonstrated by the groups of Kuwana and Eshhar in the late 1980s in the first generation CARs [18, 77]. Since this first discovery, CAR therapy has undergone major improvements and thus far most research has been performed in hematological malignancies such as B cell lymphoma and leukemia. The co-receptor CD19 showed to be an optimal target [78–80] as it is expressed early during B cell development and expression is maintained until plasma cell differentiation. B cell malignancies originating from these B cell differentiation states also express CD19. As CD19 is also expressed on normal B cells, treatment with CD19 CAR T cells will result in a transient or lasting B cell aplasia and hypogammaglobulinemia [81]. In 2003, the group of Sadelain at the Memorial Sloan-Kettering Cancer Center (New York, US) were the first to show successful transduction of peripheral blood lymphocytes with CD19 CARs in immunodeficient mice with various B cell malignancies resulting in tumor reduction and even long-term eradication [82].

The engineering of CARs has evolved over time and resulted in four generations of CAR molecules. In 1993, first-generation CAR consisted of a scFv and intracellular CD3ζ domain which mediated the production of IL-2 and non-MHC-restricted cell lysis upon activation in murine models [83]. However, the presence of costimulatory signals lead to better T cell activation (by providing signal two) and resulted in better T cell proliferation [84]. These second and third generation CARs additionally contained costimulatory domains to enhance T cell survival, activation and expansion [20, 85, 86]. Second generation CARs carry the costimulatory domains CD28 [87] or 4-1BB [85]. These showed enhanced TCR signaling, production of cytotoxic cytokines such as IL-2, proliferation and survival [20, 85, 87, 88]. Third generation CARs aim to encompass the signaling capacity of two costimulatory domains, mostly CD28 in combination with 4-1BB or OX-40. Addition of proliferative cytokines such as IL-12 or costimulatory ligands such as 4-1BBL have proven to further potentiate the anti-tumor capacity of second generation CAR T cells in preclinical studies and are currently known as the fourth generation CAR T cells [89, 90]. These CAR T cells can also be referred to as T cells redirected for universal cytokine killing (TRUCKs), which can deliver a transgenic product to the targeted tissue. By using nuclear factor of activated T cells (NFAT) to induce cytokines such as IL-12, the area around the CAR-targeted tissue is made more favorable for an immune response [91].

In xenograft models comparing the efficacy of different CAR constructs, CARs consisting of two signaling domains (CD3ζ plus CD28) and 4-1BB ligand showed the greatest anti-tumor efficacy and also increased persistence in the peripheral blood compared to first generation CAR constructs [92]. With second generation CARs, complete response rates of around 40% have been demonstrated in acute lymphoblastic leukemia (ALL) murine models treated with 5–10 × 10^6^ CD19 CD28 or 4-1BB CAR T cells [82, 93, 94]. The first clinical trial to show clinically significant responses in patients with ALL was performed by Sadelain and co-workers in 2013. In this trial, five patients with relapsed B-ALL not previously treated with allogenic hematopoietic stem cell transplantation (allo-HSCT) were treated with 1.5–3 × 10^6^/kg CD19 CD28 CAR T cells after prior conditioning treatment with cyclophosphamide (1.5–3.0 g/m^2^) and subsequent allo-HSCT as per protocol and complete remissions were seen in all treated patients (*n* = 4) [26]. Finalization of this clinical trial in 2014 with a total of 16 patients resulted in a complete response rate of 88% [95]. In a case series by Grupp et al., two children with relapsed and refractory pre-B cell ALL received 1.4 × 10^6^–1.2 × 10^7^/kg CD19 4-1BB CAR T cells and both patients showed a complete remission, one of which was ongoing 11 months post-treatment (current status unknown) [80]. In a following phase I dose-escalation trial, 21 patients with relapsed or refractory ALL or non-Hodgkin lymphoma (NHL) were either treated with 1 × 10^6^/kg/dose, 3 × 10^6^/kg/dose or the entire CD19 CAR T cell product if the product did not meet the required dosage amounts. The maximum tolerated dose was 1 × 10^6^/kg/dose, all toxicities were temporary and a complete response rate of 67% (14/21 patients) was reached [96]. More recently in 2017, patients with refractory diffuse B cell lymphoma, primary mediastinal B cell lymphoma, or transformed follicular lymphoma were treated in a multicenter phase II trial with 2 × 10^6^/kg CD19 CD28 CAR T cells following low dose preconditioning regimens with cyclophosphamide (500 mg/m^2^/day) and fludarabine (30 mg/m^2^/day) for 3 days. Of 101 treated patients, an objective response was seen in 82% and 54% of patients showed a complete response, of which 40% were durable complete responses [97]. These response rates were reproducible, as in another study complete response rates of 57% were seen in 28 patients with refractory B cell lymphoma treated with a median of 5.79 × 10^6^/kg/dose CD19 4-1BB CAR T cells [98].

As in ACT with TIL, preconditioning lymphodepletion is commonly used in the clinical treatment protocol with CAR therapy. When patients with chemotherapy-refractory chronic lymphocytic leukemia were treated solely with CAR T cells (without lymphodepleting regimen), no clinical benefit and less persistence of the CAR T cells was observed. However, it is important to note that these patients in this small study also received a lower dose of CAR T cells [78]. The clinical successes with CAR T cell therapy has recently led to the FDA approval of two CD19 CAR therapies for ALL and NHL in 2017, namely axicabtagene ciloleucel (Yescarta) with costimulatory molecule CD28 and tisagenlecleucel (Kymriah) with costimulatory molecule 4-1BB [99]. Although high complete remission rates have been demonstrated with the use of CD19 CAR T cells, resistance via the loss of CD19 has been observed in 28% of young adult and pediatric patients with acute leukemia in an international trial [100].

As stated above, most of the research with CAR T cell therapy has been performed in hematological malignancies, but also other B cell lineage-restricted targets like CD22 and B cell maturation antigen (BCMA) are currently under investigation. Moreover, CAR T cell technology is being explored in solid tumors, however achieving limited clinical activity thus far. For example in sarcomas targeting ERBB2/HER2 [101], renal cell cancer targeting carbonic anhydrase IX (CAIX) [102], non-small cell lung cancer and cholangiocarcinoma targeting epidermal growth factor (EGFR) [103], and neuroblastoma targeting GD2 [104] and other solid tumors (targeting shared antigens including mesothelin and CEA) [105]. Recently, CAR T cells directed against the colorectal cancer antigen GUCY2C were investigated in murine models showing increased antigen-dependent T cell activation, cytokine production and killing of GUCY2C-expressing tumor cells [106].

## Toxicity

The toxicity observed with ACT can grossly be divided in three groups: toxicity due to the lymphodepleting preparative regimen, immune-related toxicity and cytokine-related toxicity. During treatment with TIL, toxicities are predominantly caused by the lymphodepleting preparative regimens, resulting in pancytopenia and febrile neutropenia, and the supportive IL-2 infusions [8, 107, 108]. These toxicities are also seen in TCR gene therapy [64] and CAR therapy [97]. The most prevalent side effects seen in TIL treatment due to IL-2 include chills, high fever, hypotension, oliguria, and edema due to the systemic inflammatory and capillary leak syndrome effects and can usually be treated with supportive measures [109]. Reports have been published of autoimmune phenomena such as vitiligo or uveitis, however, these side effects are not frequently seen and uveitis usually responds well to local corticoid treatment [108].

The potency of TCR therapy lies with the recognition of antigens on tumor cells, often not tumor-specific and thus also expressed on healthy tissues. This can lead to the occurrence of “on-target, off-tumor” toxicity [36]. Targeting melanoma differentiation antigens such as MART-1 and gp100 can result in severe skin rash, uveitis, and ototoxicity due to expression of these antigens in these organs [16] and these effects seem to be dose-dependent [110]. Some of the used TCR, such as NY-ESO-1 and MAGE-A3 have undergone affinity maturation in conducted clinical trials to increase the affinity of the TCR for the target MHC-peptide complex [64, 66]. This process may increase the chance of cross-reactivity to other targets as well, and may have caused some of the severe toxicities that were seen thus far with TCRs directed at C/T antigens. The C/T antigen MAGE-A3 is not known to be present in normal tissue, but two separate TCR-based therapies directed to MAGE-A3 did result in fatal neurotoxicity and cardiotoxicity. Neurological complications may be due to cross-reactivity to MAGE-A12 which is expressed in a subset of neurons in the brain and the cardiac events may be due to cross-recognition of the muscle protein Titin [66, 111], perhaps as a result of TCR affinity enhancement. On the other hand, treatment with the C/T antigen NY-ESO-1 specific TCR transduced T cells so far seem to be safe [64]. B cell aplasia is an expected “on target off tumor” side effect of CD19 CAR T cell therapy and can be managed with replacement therapy with intravenous immunoglobulin [79].

Another common toxicity seen in ACT is cytokine release syndrome (CRS). CRS is a non-antigen-specific adverse event and is a result of activation most probably of the infused T cells through antigen recognition, leading to massive cytokine release, including IFN-γ, IL-1, and IL-6 [112]. Toxicity from CRS has a very heterogeneous presentation, but usually involves fever, hypotension, tachycardia, and respiratory insufficiency and it is potentially fatal, as described by van den Berg et al. in a case report of a patient with metastatic melanoma treated with a TCR recognizing MART-1 [110]. The severity of CRS is correlated with tumor burden [26, 113]. Severe and life-threatening CRS can effectively be treated with tocilizumab (human monoclonal antibody against the IL-6 receptor) or siltuximab (monoclonal antibody against IL-6) and sometimes corticosteroids [112, 114, 115]. Additional targeted immunosuppressive agents like infliximab (anti-TNF-α antibody) and anakinra (anti-IL-1R antibody) have been used in some very severe cases [112], the latter of which has recently also been shown to be effective against neurotoxicity in CAR T cell therapy in a murine model [116].

Neurological complications, including confusion, delirium and occasional seizures and cerebral edema, occasionally resulting in death, have been observed with the use of CD19-specific CAR T cells [117]. The underlying pathogenesis of this neurotoxicity remains currently unknown. Recently, endothelial cell activation and increased blood-brain barrier permeability were found to play a role at the initiation of this toxicity [118]. Intensive research is currently ongoing to further elucidate the underlying pathogenesis of both CRS and the neurotoxicity, identify useful biomarkers and optimize current treatment algorithms [119].

Lastly, an IgE-mediated anaphylactic reaction has been observed in a patient treated with autologous T cells electroporated with mRNA encoding for a CAR derived from a murine antibody to mesothelin [120]. This reaction may have been induced by “foreign” CAR moieties and current strategies for CAR therapy involve the use of humanized or even fully human scFv to circumvent these IgE-mediated responses.

## Future prospective of adoptive cell therapy and conclusions

The ultimate goal of adoptive cell therapy for malignancies is to create an optimized personalized cellular product solely reactive to the tumor. In the past decades, the production of TIL, TCRs, and CARs have all undergone developments to improve its efficacy as anti-cancer treatment. TIL are a heterogeneous cell product and the presence of antigen-reactive TIL is a key determinant of anti-tumor reactivity [121]. Enrichment for tumor-reactive TIL through CD137 [122] or PD-1 [123] selection is currently being investigated as a method to increase the anti-tumor reactivity.

Genetic editing of TIL may also improve its functionality, as has already been demonstrated with Zinc finger nuclease engineering which decreases the PD-1 expression in TIL, improving the efficacy [124].

In TCR therapy, it is of major importance that targetable antigens are identified which are expressed on the tumor, but not on healthy tissue to decrease the “on-target, off-tumor” toxicity and to further potentiate its effector function. Currently, oncogenes such as BRAF^V600E^ driver mutations are also being explored as possible therapeutic targets for ACT and have already shown clinical activity in a patient with advanced melanoma [125]. HPV antigens E6 and E7 are being explored as targets for ACT as treatment of HPV-associated cancers [10, 69]. Neo-antigens that arise as a result of tumor-specific mutations appear exquisite targets for TCR gene therapy as these antigens are, like viral antigens, fully foreign to the immune system. Gene therapy targeting these antigens are also being developed.

As in ACT with TCR gene therapy, the main challenges in CAR therapy comprise new target discovery, reduction of toxicity, and improvement of cell trafficking. Because of the correlation between tumor burden and toxicity from CAR T cell therapy [26, 113], tumor reduction before therapy or dose adaptation strategies could possibly be applied to reduce side effects. More promising are current investigations with CAR T cells engineered to contain suicide genes or switches (for example iCasp9) which are evaluated in preclinical and clinical studies [126]. CAR T cells seem to express inhibitory receptors such as PD-1 in an exhausted state, which upregulates PD-L1 on tumor cells. Combinatorial approaches of CAR T cell therapy and PD-1 blockade have resulted in improved CAR T cell activity and tumor eradication in preclinical experiments and clinical studies are currently ongoing [127, 128]. Furthermore, the combination of CAR T cell therapy with oncolytic virus-driven production of a bispecific T cell engager showed enhanced efficacy in a mouse model compared to both monotherapies [129].

In conclusion, ACT represents a personalized immunotherapeutic approach that has developed rapidly in recent years. Great successes have already been seen with the use of TIL treatment in melanoma and CAR therapy in hematologic malignancies. However, further optimization of this promising treatment modality is warranted to enhance the anti-tumor effect and reduce the associated toxicity. More than 100 clinical trials have been initiated since 2015 and are currently being conducted with ACT [27] and should provide new insights in the efficacy and further developments of this treatment modality.

## Data Availability

Not applicable. All cited published original research in this manuscript are publically available.

## Abbreviations

ACT: Adoptive cell therapy
ALL: Acute lymphoblastic leukemia
BCMA: B cell maturation antigen
BRAF: B-Raf
C/T: Cancer/testis
CAIX: Carbonic anhydrase IX
CAR: Chimeric antigen receptor
CD: Cluster of differentiation
CEA: Carcinoembryonic antigen
CRS: Cytokine release syndrome
EGFR: Epidermal growth factor receptor
ERBB2: erb-b2 receptor tyrosine kinase 2
FDA: Food and Drug Administration
GMP: Good manufacturing practice
gp100: Glycoprotein 100
GUCY2C: Guanylate cyclase 2C
Gy: Gray
HD: High dose
HER2: Human epidermal growth factor receptor 2
HLA: Human leukocyte antigen
HPV: Human papillomavirus
HSCT: Hematopoietic stem cell transplantation
i.v.: Intravenous
iCasp9: Inducible caspase-9
IFN: Interferon
IL: Interleukin
IMC: Immunocore
IU: International unit
kg: Kilogram
LAK: Lymphokine-activated killer
m: Meter
MAGE: Melanoma antigen gene
MART-1: Melanoma antigen recognized by T cells 1
mg: Milligram
MHC: Major histocompatibility complex
NFAT: Nuclear factor of activated T cells
NHL: Non-Hodgkin lymphoma
NIH: National Institutes of Health
NMA: Non-myeloablative
PD-1: Programmed cell death protein-1
PD-L1: Programmed death ligand-1
REP: Rapid expansion protocol
SB: Surgery branch
scFv: Single-chain variable fragment
SSX: Synovial sarcoma X breakpoint
TBI: Total body irradiation
TCR: T cell receptor
TIL: Tumor-infiltrating lymphocytes
TNF: Tumor necrosis factor
TRUCK: T cells redirected for universal cytokine killing
US: United States
